# Identification of a Transcription Factor Signature That Can Predict Breast Cancer Survival

**DOI:** 10.1155/2021/2649123

**Published:** 2021-02-19

**Authors:** Chunni Fan, Jianshi Du, Ning Liu

**Affiliations:** ^1^Department of Breast Surgery, The Third Hospital of Jilin University, Changchun, Jilin 130033, China; ^2^Department of Vascular Surgery, The Third Hospital of Jilin University, Changchun, Jilin 130033, China

## Abstract

**Background:**

The expression pattern of transcription factors (TFs) can be used to develop potential prognostic biomarkers for cancer. In this study, we aimed to identify and validate a TF signature for predicting disease-free survival (DFS) of breast cancer (BRCA) patients.

**Methods:**

Lasso and the Cox regression analyses were applied to construct a TF signature based on a gene expression dataset from TCGA. The prognosis value of the TF signature was investigated in the TCGA database, and its reliability was further validated in 3 independent datasets from Gene Expression Omnibus (GEO). The prognosis performance of the TF signature was compared with 4 previously published gene signatures. To investigate the association between the TF signature and hallmarks of cancer, Gene Set Enrichment Analysis (GSEA) was carried out. The correlations of the TF signature and the levels of immune infiltration were also investigated.

**Results:**

An 11-TF prognostic signature was constructed with good survival prediction performance for BRCA patients. By using the risk score model based on the 11-TF signature, BRCA patients were stratified into low- and high-risk groups and showed good and poor disease-free survival (DFS), respectively. The risk score was an independent prediction indicator when adjusting for other clinicopathological factors. Furthermore, the 11-TF signature had a better survival prediction performance compared to 4 previously published gene signatures. Moreover, the risk score was a cancer hallmark. Finally, a high-risk score was associated with higher infiltration of M0 and M2 macrophages and was associated with a lower infiltration of resting memory CD4^+^ T cells and CD8^+^ T cells.

**Conclusion:**

The findings in this study identified and validated a novel prognostic TF signature, which is an independent biomarker for the prediction of DFS in BRCA patients.

## 1. Introduction

Breast cancer (BRCA) is one of the leading causes of death from cancer in women and represents a heterogeneous group of neoplasms originating from the epithelial cells lining the milk ducts [1, 2]. Traditionally, tumor size; status of nodal metastasis; and status of the estrogen receptor (ER), progesterone receptor (PR), and HER2 were taken as useful prognostic biomarkers for BRCA in the clinic [3–5]. However, these prognostic biomarkers are still limited in accurately predicting survival due to the genetic heterogeneity of BRCA patients [6]. Therefore, establishing an accurate and robust signature to guide prognostic stratification for BRCA is of considerable importance.

Transcription factors (TFs) are DNA-binding proteins that bind to the promoter sequences of genes and subsequently regulate gene expression [7]. Previous studies have demonstrated that TFs are involved in several key cellular processes, such as cell growth, differentiation, proliferation, and cell death [8–10]. TFs are often aberrantly expressed in patients with BRCA, and the association between the expression of TFs and patient survival has been demonstrated in BRCA [11]. Nevertheless, the expression pattern and prognostic ability of TFs in BRCA were investigated only in a few studies.

In the present study, we identified several survival-related TFs and developed an 11-TF signature for the prediction of the disease-free survival (DFS) of BRCA patients by analyzing the gene expression and the corresponding clinical data of BRCA patients from The Cancer Genome Atlas (TCGA). The prognosis performance of the TF signature was validated in 3 independent datasets from Gene Expression Omnibus (GEO) and compared with 4 previously published gene signatures. In addition, the association between the risk score based on the 11-TF signature and cancer hallmarks, and immune cell infiltration was investigated.

## 2. Materials and Methods

**Figure 1:**
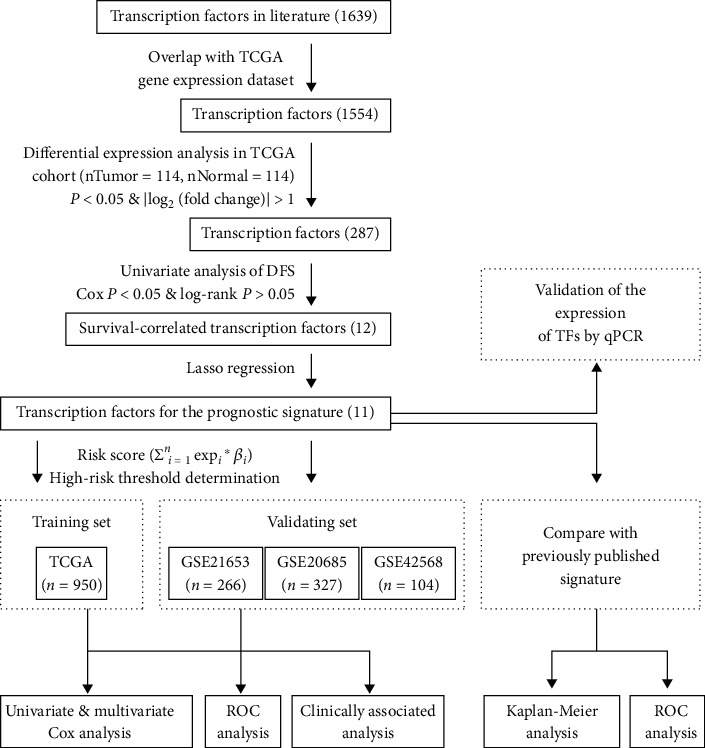
Flow chart of the study.

All data analyses were performed using R software (http://www.r-project.org/version 3.5.1). A flowchart of the study process is shown in Figure 1.

### 2.1. Data Collection

In 2018, Lambert et al. published a compelling review covering ≥1,600 likely human TFs underlying human physiology and disease [7]. In this study, we collected 1639 TFs from the literature for further exploration. Gene expression data from BRCA patients were downloaded from the TCGA through the University of California Santa Cruz Xena Browser (UCSC Xena, http://xena.ucsc.edu/) [12]. The overlap between TFs in the TCGA and the 1639 TFs were extracted, and 1554 TFs were obtained for differential expression analysis in the TCGA cohort (114 paired normal and tumor samples).

A total of 950 patients with corresponding genomic data (read counts) and clinical information including DFS data were recruited from the TCGA database, which served as training sets. Gene expression profiles and corresponding clinical information of BRCA patients were selected from the online public Gene Expression Omnibus (GEO, http://www.ncbi.nlm.nih.gov/geo/) database, including three independent datasets with accession numbers GSE20685 [13], GSE21653 [14, 15], and GSE42568 [16]. GSE20685 consisted of 327 BRCA cases, GSE21653 was composed 266 BRCA patients, and GSE42568 contained 121 samples (104 BRCA patients and 17 normal controls, Table 1). A total of 697 tumor samples were generated using the same chip platform (GPL570 Affymetrix Human Genome U133 Plus 2.0 Array), and these datasets served as validation sets.

### 2.2. Tumor Samples

Tumor samples and matched adjacent normal tissues were collected from 20 BRCA patients at the Third Hospital of Jilin University (Jilin, China), between January 2020 and November 2020. Oral consent was obtained from all patients. All experiments were conducted according to the ethics approval from the Ethics Committee of The Third Hospital of Jilin University (Jilin, China). All samples were anonymized before analysis to guarantee the protection of privacy.

### 2.3. Identification of Differentially Expressed TFs

The Limma package was used to identify differentially expressed TFs between the 114 pairs of BRCA and normal control samples [17]. Differentially expressed TFs were detected with the cutoff criteria of ∣log_2_fold change (FC) | >1 and adjusted *P* values ≤ 0.05. All *P* values were adjusted using the Benjamini and Hochberg method to control false discovery rates (FDR) [18]. Volcano plots were created based on the ggplot2 in R package (version 3.2.0, https://cran.r-project.org/web/packages/ggplot2/index.html) [19]. Hierarchical cluster analysis on the expression of differential TFs was performed using the pheatmap package (version 1.0.12, https://cran.r-project.org/web/packages/pheatmap/index.html) [20].

### 2.4. Construction of the Prognostic TF Signature

To determine prognostic TF candidates, univariate regression analysis was performed to assess the correlation between differentially expressed TFs and DFS in the BRCA cohort. Only TFs with the Cox *P* < 0.05 and log-rank *P* < 0.05 were deemed as correlating to survival. Least Absolute Shrinkage and Selection Operator (Lasso) regression models have considerable advantages in terms of sensitivity and specificity, and their coefficients can be used to determine true regulatory efficacies in tissues [21]. In this study, Lasso regression analysis was employed to determine whether the candidates were efficient variables for DFS, and those with coefficients = 0 were eliminated. Signature-based prognostic models were used to compute a risk score for each patient using

(1) $$\begin{array}{c} \text{Risk score}=\overset{n}{\underset{i=1}{\sum }}E_{i}*\beta_{i}, \end{array}$$

where *n* is the number of selected genes, *E*_*i*_ represents the expression of each gene, and *i* and *β*_*i*_ represent the coefficient of gene *i*. Of note, the average risk score was defined as the cutoff criteria to classify samples into the high-risk (scores ≥ median values) and low-risk (scores ≤ median values) groups.

For further validation of the prognostic performance of the TF signature, the AUC of time-dependent receiver operating characteristic (ROC) curves was assessed [22]. Defining points were set as 1-, 2-, 3-, 4-, 5-, and 10-year time-dependent ROC curves employed to assess the predictive value of the risk score for time-dependent outcomes [22]. A higher AUC indicated a higher sensitivity.

### 2.5. Gene Set Enrichment Analysis (GSEA) of the TF Signatures and Cancer Hallmarks

Cancer hallmarks summarize the relevant information from the datasets, thereby reducing both variation and redundancy. This provides more refined and concise inputs for GSEA analysis [23]. In total, 50 hallmark gene sets were recruited from the molecular signature database (MSigDB, http://software.broadinstitute.org/gsea/msigdb), which were imported into the GSEA (http://software.broadinstitute.org/gsea/index.jsp).

### 2.6. Quantitative Real-Time PCR (qRT-PCR)

To validate the mRNA expression levels of the TFs between tumor and normal breast tissue samples from the TCGA dataset, mRNA expression levels of these TFs were measured by qRT-PCR in collected fresh frozen tumor tissue samples. Subsequently, qRT-PCR was implemented on RNA collected from cancer and normal lysates. In brief, total RNA was separated by means of FastPure Cell/Tissue Total RNA Isolation Kit V2 (Vazyme Biotech Co., Ltd., Nanjing, China) based on the manufacturer's guidelines. Next, RNA was transcribed to cDNA using a HiScript II 1st Strand cDNA Synthesis Kit. All reactions were implemented using an AceQ Universal SYBR qPCR Master Mix (Vazyme Biotech Co., Ltd., Nanjing, China) on a QuantStudio™ 5 Real-Time PCR System (ABI, Life Technologies, Thermo Fisher Scientific, Waltham, MA, USA). The fold change in expression was computed as the delta-delta threshold cycle (ΔΔCt) after normalization to an internal reference. The primer pairs for qRT-PCR are shown in Table S1.

### 2.7. Deconvolution of the Infiltrated Immune Cells in the Tumor Microenvironment

Cell type Identification By Estimating Relative Subsets Of RNA Transcripts (CIBERSORT) is an algorithm that is used to characterize the cell composition of complex tissues based on gene expression profiles [24]. In the present study, the CIBERSORT method was applied to investigate the immune cell infiltration level of tumor samples from the TCGA database.

### 2.8. Statistical Analysis

Survival estimates were performed using the Kaplan-Meier method [25]. The DFS was defined as the time between diagnosis and disease recurrence. Statistical differences between survival times were assessed using log-rank tests [26]. Univariate and multivariate regression analyses were performed using the Cox proportional hazards model [27]. *P* ≤ 0.05 was considered statistically significant.

## 3. Results

### 3.1. Identification of a Prognostic TF Signature

**Figure 2:**
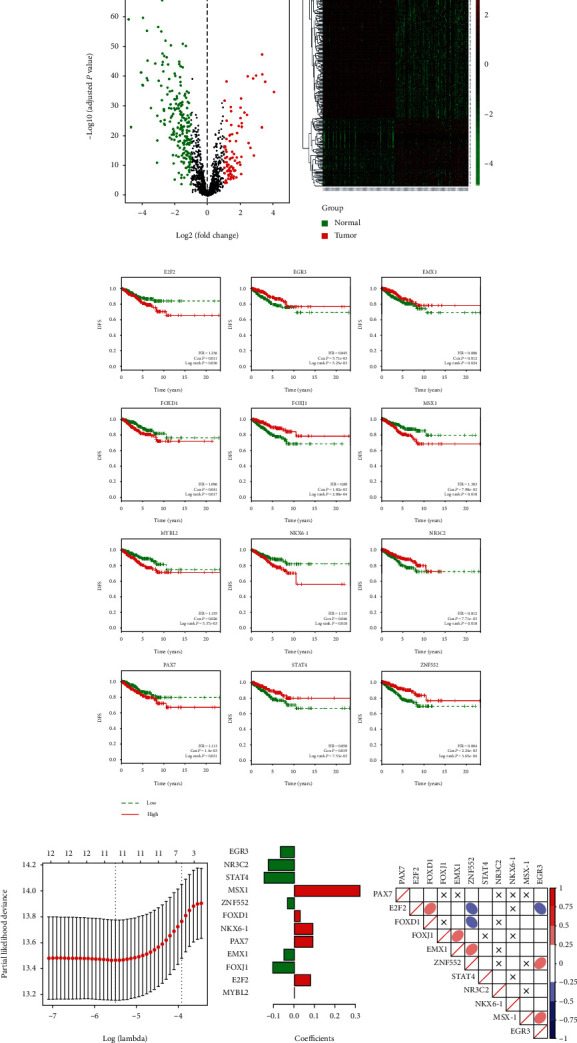
Identification of the prognostic 11-transcription factor (11-TF) signatures. (a) Volcano plots for differentially expressed TFs. (b) Heatmaps for differentially expressed TFs. (c) Disease-free survival (DFS) curves for the 12 survival-correlated TFs in the high-risk and low-risk groups. (d) Lasso regression analysis for the 12 TF candidates. (e) Coefficients obtained from the Lasso algorithm for the 12 TF candidates. (f) Spearman correlation analysis of the prognostic 11-TF signatures for the prediction of DFS of breast cancer (BRCA). X: *P* > 0.05. Color bars: Spearman's coefficients (*r*).

In this study, a total of 287 differentially expressed TFs were identified between BRCA and normal controls under the threshold of ∣log_2_FC | >1 and *P* < 0.05. Of the 287 TFs, 85 were upregulated and 202 were downregulated. Distribution of the differentially expressed TFs was depicted using volcano plots in which the log_2_ (FC) was the abscissa and the –log_10_FDR was the ordinate (Figure 2(a)). Heatmap visualization of these differentially expressed TFs is presented in Figure 2(b) and shows that BRCA (Tumor) and normal controls (Normal) could be well separated by these TFs (Figure 2(b)).

Univariate regression analysis on the DFS led to the extraction of 12 survival-associated TFs as candidate prognostic signatures, including E2F2, EGR3, EMX1, FOXD1, FOXJ1, NKX6-1, NR3C2, PAX7, STAT4, MYBL2, ZNF552, and MSX1. As shown in Figure 2(c), higher expression of E2F2, FOXD1, MSX1, MYBL2, NKX6-1, and PAX7 was associated with a poorer DFS, while elevated expression of EGR3, EMX1, FOXJ1, NR3C2, STAT4, and ZNF552 was associated with improved DFS. To identify a prognostic TF signature, Lasso regression analysis was performed for the 12 prognosis-associated TFs in the TCGA datasets (Figure 2(d)). As shown in Figure 2(e), 11 TFs were selected to construct the prognostic TF signature, while MYBL2 with the regression coefficient of 0 was removed. General information of the 11 TFs is shown in Table 2. Spearman's correlation analysis suggested that expression of the identified TFs that composed the TF signature showed little to no correlation, thereby indicating an independent prediction power of each TF (Figure 2(f)).

### 3.2. The Risk Score Based on the 11 TF Signatures Predicts the Survival of BRCA Patients

**Figure 3:**
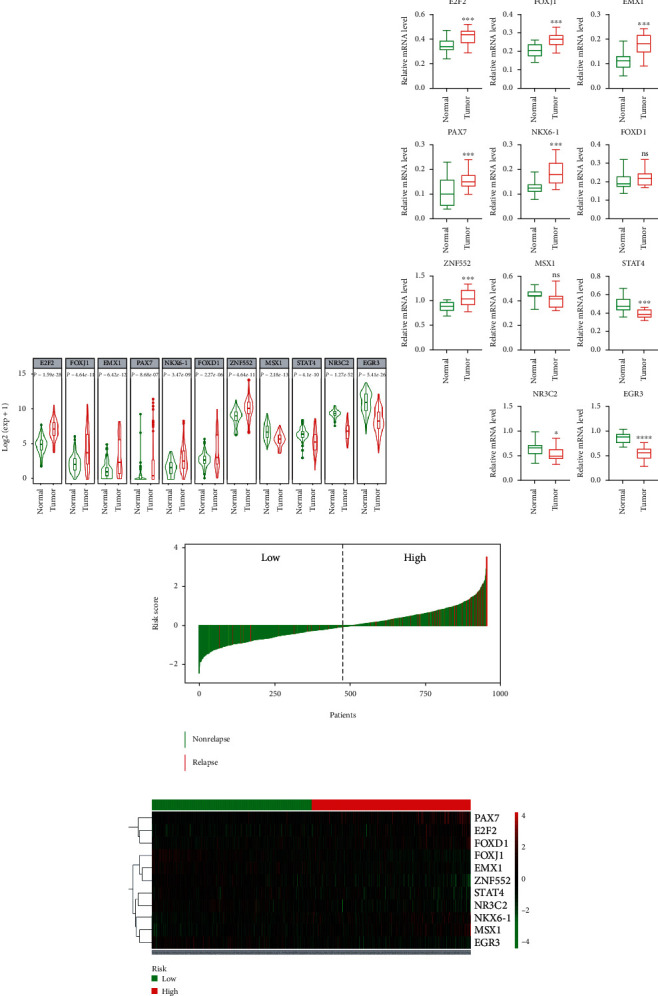
TF expression levels, patient relapse status, and cluster performance. (a) Expression patterns of the 11 TF prognostic signatures between BRCA patients (Tumor) and normal controls (Normal) in the TCGA database. (b) Experimental verification for mRNA expression levels of the 11 TF signatures between BRCA patients (Tumor) and normal controls (Normal). (c) Relapse status of BRCA patients in high-risk and low-risk groups. (d) The hierarchical cluster performance of the 11 prognostic TFs between high-risk and low-risk groups.

We next analyzed the expression pattern of the 11 TFs in the TCGA database. Compared to normal samples, the expression of E2F2, FOXJ1, EMX1, PAX7, NKX6-1, FOXD1, and ZNF552 was significantly higher (*P* < 0.05) and the expression of MSX1, STAT4, NR3C2, and EGR3 was significantly lower in tumor samples (all *P* < 0.05) (Figure 3(a)). Consistent with these results, qRT-PCR confirmed that the mRNA expression level of these TFs showed similar trends to their expression pattern in the TCGA database (Figure 3(b)). Subsequently, a risk score model was constructed and used to predict the prognosis of BRCA patients. According to the median risk score, BRCA patients were divided into low-risk and high-risk groups (Figure 3(c)). Patients with high-risk scores were more likely to relapse compared to patients with low-risk scores (Figure 3(c)). Hierarchical cluster performance showed that the 11 prognostic TFs were well classified between low-risk and high-risk groups (Figure 3(d)).

**Figure 4:**
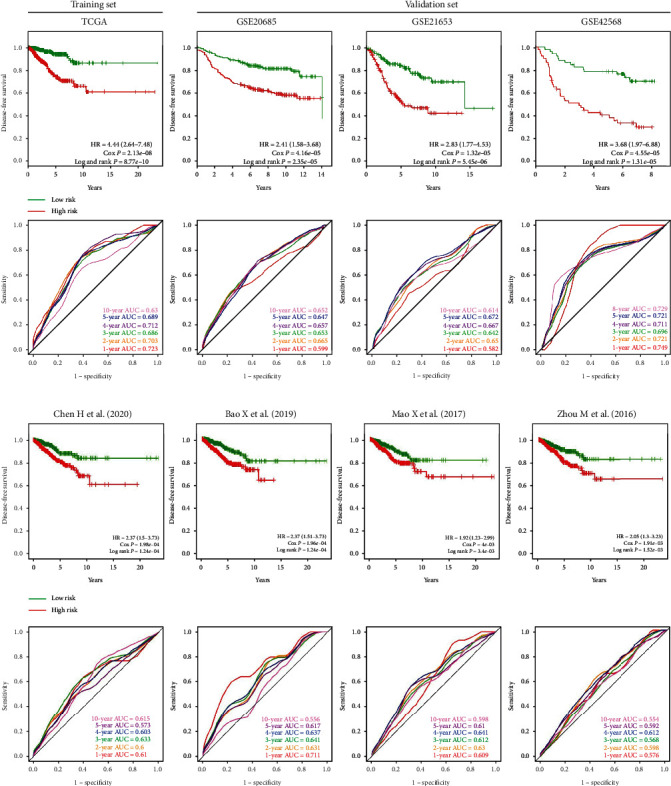
The Kaplan-Meier and receiver operating characteristic (ROC) curves of BRCA patients stratified by risk score. (a) The Kaplan-Meier and ROC curves of BRCA patients stratified by risk score in training datasets (TCGA) and validation datasets (GSE20685, GSE21653, and GSE42568). (b) The Kaplan-Meier curves and ROC curves of previously published prognostic signatures.

Next, we analyzed the survival prediction power of the risk score in the TCGA database, which was regarded as the training set. As shown in Figure 4(a), patients in the low-risk group had significantly longer DFS compared to patients in the high-risk group (HR = 4.44, 95%CI = 2.64‐7.48, the Cox *P* = 2.13*e* − 08, and log-rank *P* = 8.77*e* − 10). Next, the predictive performance of the risk score was validated in 3 GEO datasets and showed similar prediction values (GSE20685: HR = 2.41, 95%CI = 1.58‐3.68, the Cox *P* = 4.16*e* − 05, and log-rank *P* = 2.35*e* − 05; GSE21653: HR = 2.83, 95%CI = 1.77‐4.53, the Cox *P* = 1.32*e* − 05, and log-rank *P* = 5.45*e* − 06; and GSE42568: HR = 3.68, 95%CI = 1.97‐6.88, the Cox *P* = 4.55*e* − 05, and log-rank *P* = 1.31*e* − 05).

To assess the sensitivity and specificity of the 11-TF signature, time-dependent ROC curves for DFS predictions were constructed (Figure 4(a)). Excluding the 1-year AUC values in GSE20685 and GSE21653, all other AUC values were ≥0.6. The 11-TF signature achieved AUC values for 5-year survival of 0.689, 0.647, 0.672, and 0.721 in TCGA, GSE20685, GSE21653, and GSE42568, respectively. These results suggested that the risk scores based on the 11-TF signature were effective for the prediction of the DFS of BRCA patients and had a robust predictive performance.

### 3.3. The 11-TF Signature Showed Superior Prediction Performance Compared to Previously Published Prognostic Signatures

In this study, our 11-TF signature was compared with 4 previously published prognostic signature panels: the 9-TF signature reported by Chen et al. [28], the 12-lncRNA signature by Zhou et al. [29], the 13-gene epigenetic signature by Bao et al. [30], and the 12-gene prognostic signature by Mao et al. [31]. As shown in Figure 4(b), all patients in the low-risk group had a longer DFS compared to patients in the high-risk group (Chen et al.: log-rank *P* = 1.24*e* − 04; Bao et al.: log-rank *P* = 1.24*e* − 04; Mao et al.: log-rank *P* = 3.4*e* − 03; and Zhou et al.: log-rank *P* = 1.52*e* − 03). When compared to the 4 previously published gene signatures, our 11-TF signature obtained a smaller log-rank *P* value. Moreover, our 11-TF signature had the highest 1–10-year AUC values. Accordingly, these findings highlighted the best performance of our 11-TF signature.

### 3.4. The Risk Score Is an Independent Prognostic Factor

Here, we firstly performed univariate regression analysis to investigate the correlation between the clinical and pathological factors and the DFS of BRCA patients. As shown in Table 1, the risk score, pathological stage, ER status, and PR status were significantly correlated to the DFS of patients with BRCA in the TCGA database; the risk score, T stage, N stage, M stage, and adjacent CT were associated with the DFS of patients in GSE20685; only risk scores and T stage were associated with the DFS of BRCA patients in GSE21653; whereas risk scores, N stage, and ER status were related to the DFS of patients in GSE42568. Interestingly, we found that the risk score was associated with DFS across the entire BRCA cohort. Subsequently, multivariate regression analysis was performed to investigate which factors were independent prognostic factors. As shown in Table 1, only the risk score was an independent prognostic factor across all datasets.

**Figure 5:**
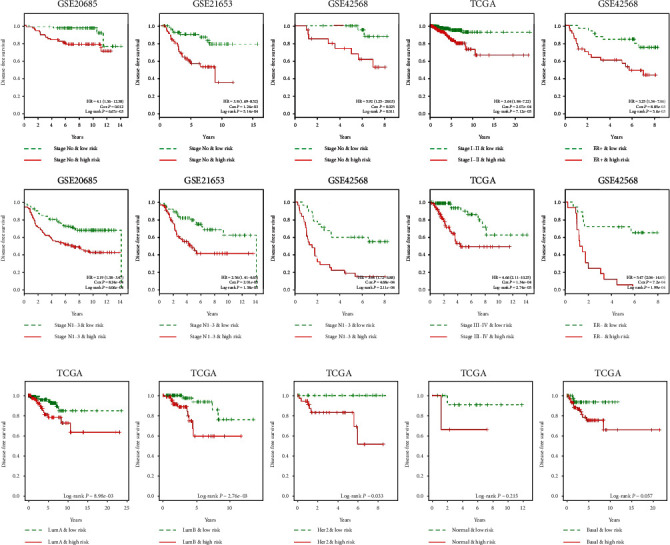
The Kaplan-Meier and ROC curves of BRCA patients stratified by clinicopathological factors, molecular subtypes, and risk score. (a) The Kaplan-Meier and ROC curves of BRCA patients stratified by clinicopathological factors and risk score. (b) The Kaplan-Meier and ROC curves of BRCA patients stratified by molecular subtypes and risk score.

We further performed stratification analysis of patients with clinicopathological variables, molecular subtype, and the risk score. As shown in Figure 5(a), BRCA patients in the low-risk group had longer survival times compared to those in the high-risk groups for all stratified clinicopathological variable subgroups in each dataset. Specifically, comparison of all curves between high- and low-risk groups suggested significant differences with log-rank *P* < 0.05 and the Cox *P* < 0.05. Moreover, stratification analysis of molecular subtypes demonstrated that high-risk patients showed poor survival trends in all molecular subtypes (Figure 5(b)). However, no differences were observed for DFS for normal, and basal subtypes between high- and low-risk groups. Taken together, these data suggested that the risk score was an independent prognostic factor for the predictions of the DFS of BRCA patients.

### 3.5. The Risk Score Is Associated with Immune Cell Infiltration

**Figure 6:**
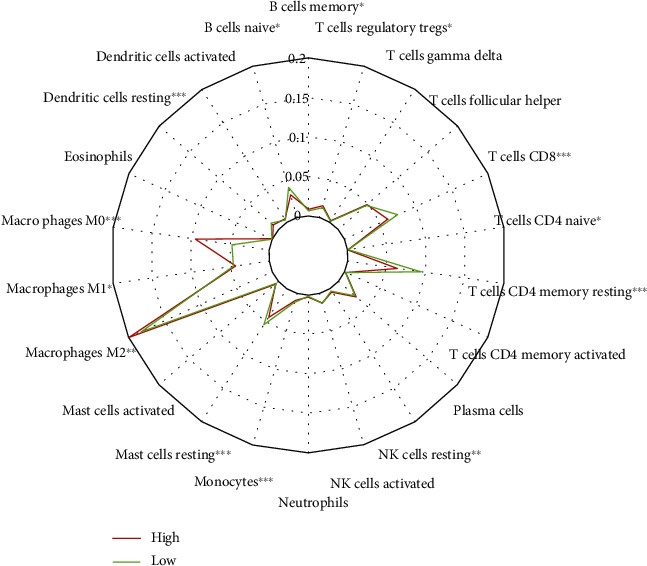
The composition of the fractions of immune cells between high- and low-risk groups.

Using the CIBERSORT method, we estimated the abundance of 22 immune cell subtypes to investigate the association between the risk score and immune cell infiltration in the tumor microenvironment. As shown in Figure 6, the fractions of M0 and M2 macrophages, resting natural killer (NK) cells, regulatory T cells, and memory B cells were significantly higher in the high-risk group, whereas native B cells, resting mast cells, monocytes, resting memory and native CD4^+^ T cells and CD8^+^ T cells, resting dendritic cells, and M1 macrophages were remarkably higher in the low-risk groups. Furthermore, the two immune cells with the highest proportion in the high-risk group were M0 and M2 macrophages. Two types of immune cells, including resting memory CD4^+^ T cells and CD8^+^ T cells had the highest proportion in low-risk patients.

### 3.6. The Risk Score Is Associated with the Hallmarks of Cancer

**Figure 7:**
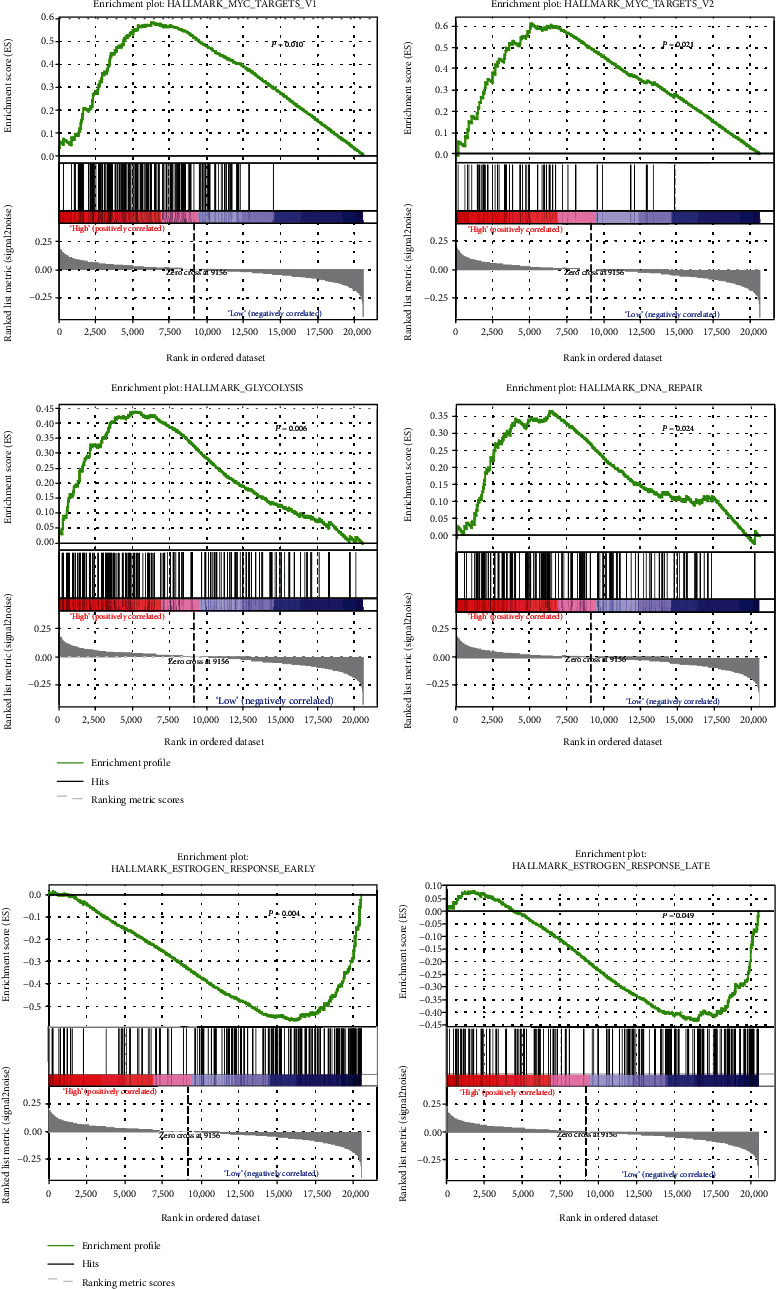
Gene Set Enrichment Analysis (GSEA) of hallmarks. (a) Four hallmarks were enriched in the high-risk group. (b) Two hallmarks were enriched in the low-risk group.

To investigate if the 11-TF signature was associated with tumor biological processes, Gene Set Enrichment Analysis (GSEA) was performed. As shown in Figure 7, a total of 6 hallmark gene sets were enriched (*P* < 0.05). Four hallmarks (including MYC_TARGETS_V1, MYC_TARGETS_V2, GLYCOLYSIS, and DNA_REPAIR) were associated with high-risk scores, suggesting that the activation of these biological processes may participate in BRCA progression. In contrast, the other 2 hallmarks (ESTROGEN_RESPONSE_EARLY and ESTROGEN_RESPONSE_LATE) were associated with low-risk scores, suggesting that their activation inhibits tumor progression and improves survival in BRCA patients.

## 4. Discussion

Because of the heterogeneous feature of BRCA, traditional factors appear not to be sufficient for predicting the survival of BRCA patients. Hence, the development of valuable biomarkers for survival-specific prognosis is of utmost importance. In recent years, several studies have determined multigene panels that might serve as prognosis indicators in BRCA. For example, Chen et al. [28] used gene expression data and clinical data from the TCGA and GEO databases to identify a 9-TF signature, which may play important prognostic roles in patients with BRCA. Sun et al. [32] analyzed lncRNA expression profiles of BRCA patients from the GEO database to identify a nine-lncRNA signature which could be a valuable prognostic biomarker to predict the metastatic risk in patients with BRCA. In another study, the lncRNA expression profiles of BRCA patients were analyzed to construct a 12-lncRNA signature to predict recurrence-free survival [29]. In 2019, Bao et al. [30] established a 13-gene epigenetic signature via combining mRNA expression and DNA methylation datasets. A 12-gene prognostic signature was identified based on the combined independent BRCA databases by means of gene coexpression network analyses [31]. Significantly, all these studies focused on the identification of prognostic signatures; however, they did not consider immune infiltration analysis nor implement qRT-PCR to verify the expression level of these signatures using frozen tissue samples. In our study, in addition to establishing and validating a prognostic model of an 11-TF signature that was significant to predict DFS in BRCA patients through integrating TF data, gene expression dataset, and clinical information, immune infiltration analysis was also performed to investigate the correlation of immune cells and our TF signature. Moreover, we verified the expression level of TFs using qRT-PCR using frozen tissue samples. Our findings showed that the risk score was an independent prognostic factor to successfully classify tumor patients into high-risk and low-risk groups with significant differences in DFS. Thus, these results indicate the reproducibility and reliability of the 11-TF signature for DFS predictions of BRCA.

The 11-TF prognostic model was comprised of E2F2, EGR3, EMX1, FOXD1, FOXJ1, NKX6-1, NR3C2, PAX7, STAT4, ZNF552, and MSX1. In a previous study, Chen et al. reported that the E2F family of TFs collectively or individually regulate cell proliferation in cancer [33]. Specifically, E2Fs have prognostic value in BRCA, which is independent of clinical parameters [34]. In a previous study, it was reported that the activation of E2F2 is associated with the resistance to antiestrogen treatment for ER*α*-positive BRCA [35]. HER2-regulated E2F2 expression further impacted cell-matrix adhesion, with potential consequences for metastatic colonization [36]. In addition, upregulated E2F2 acts as a crucial intermediate in HER2-directed signaling circuits in BRCA [37]. Malorni et al. showed that gene expression signatures could be established based on the investigation of genes correlating with E2F1 and E2F2 expression in BRCA within the TCGA database [38]. Our results are consistent with the findings in these studies, thereby further validating the key functions of E2F2 in BRCA prognosis. EGR3 actively participates in estrogen signaling [39]. Inoue et al. demonstrated that EGR3 plays a critical role in the physiology of normal and malignant mammary cells, through the induction of estrogen-responsive genes [40]. In this study, GSEA revealed that hallmarks of the estrogen response (ESTROGEN_RESPONSE_EARLY and ESTROGEN_RESPONSE_LATE) were enriched in patients in low-risk groups. We inferred that EGR3 was a suppressor of TFs through its ability to promote estrogen signaling during BRCA progression. The FOX family of DNA-binding proteins regulates DNA transcription and repair [41]. Interestingly, in this study, two members of the FOX family (FOXD1 and FOXJ1) were identified as prognostic signatures for DFS predictions in BRCA patients. FOXD1 functions as an oncogene in lung, breast, and brain cancers [42, 43] and is upregulated to promote breast cancer cell proliferation and chemoresistance by inducing G1 to S transition [42]. FOXJ1 is hypermethylated in BRCA cell lines and clinical tissue samples, revealing its role as a putative tumor suppressor gene [44]. In addition, DNA_REPAIR was a significant hallmark of cancer that was enriched in high-risk groups, which is in accordance with the functions of the FOX family, thereby verifying the consistency of our studies.

Cancer is a complex multistage process involving genetic and epigenetic changes that result in the activation of oncogenic signaling and/or the inactivation of tumor suppressor signals [45]. Cancer cells acquire a number of changes that promote tumor growth and invasion [46]. MSigDB, originally developed for GSEA, remains one of the largest and most popular repositories of gene datasets [47]. The GSEA database focuses on the coordinated differential expression of annotated groups of genes or gene sets and produces data that can be more easily interpreted in terms of relevant biological processes [48]. We utilized GSEA to select significant hallmarks of DFS predictions from MSigDB and the 11-TF prognostic signature. MYC_TARGETS_V1, MYC_TARGETS_V2, GLYCOLYSIS, and DNA_REPAIR were enriched in the high-risk group, while ESTROGEN_RESPONSE_EARLY and ESTROGEN_RESPONSE_LATE were significantly enriched in low-risk groups. MYC promotes cell cycle progression through the activation of Cdk4, Cdc25A, E2F1, and E2F2 [49]. Furthermore, reduced proliferation is driven by decreased MYC/E2F1 activity in enteroendocrine cells [50]. It had been suggested that high MYC expression is associated with high GSEA scores for both MYC hallmarks (MYC_TARGETS_V1 and MYC_TARGETS_V2) [23]. In 2019, Yu et al. showed that MYC plays a critical role in the aggressive proliferation-related phenotypes exhibited by BRCA cells expressing ER*α* mutations [51]. Thus, targeting MYC in combination with other oncogenic pathways provides a promising therapeutic strategy for BRCA [52]. In this study, the 11 TFs were combined into a single panel, and its prognostic value in DFS in BRCA was established.

Recently, in several studies, it was suggested that tumor infiltrating immune cells are correlated with the prognosis of cancer patients [53, 54]. To further determine the relationship between immune infiltrating cells and the TF signature, CIBERSORT analysis was performed to reveal the composition of immune infiltrating cells. In the present study, high infiltration of M2 macrophages was associated with a high-risk score, whereas high infiltration of resting memory CD4^+^ T cells and CD8^+^ T cells was associated with a low-risk score. These results were consistent with the biological function of these immune cells in cancer progression. In a previous study, it was revealed that M2 macrophages play important roles in enhancing tumor growth and metastasis [55]. Furthermore, M2 macrophages have been reported to be linked with unfavorable prognosis in triple-negative breast cancer patients [56]. Resting memory CD4^+^ T cells and CD8^+^ T cells have been demonstrated to be correlated with the increase of overall survival (OS) and DFS of BRCA [57]. Based on these results, we believe that the risk score of this 11-TF signature is reliable for predicting the prognosis of BRCA patients.

In conclusion, we have identified and validated a prognostic 11-TF signature for the prediction of DFS of patients with BRCA. The 11-TF signature was an independent factor and may serve as a complement prognostic factor for clinicopathological factors.

## Figures and Tables

**Table 1:** The Cox regression analysis of the association between clinicopathological factors (including risk scores) and the disease-free survival of BRCA patients.

Variables	Group	Patients (*N*)	Univariate analysis		Patients (*N*)	Multivariate analysis	
			HR (95% CI)	*P*		HR (95% CI)	*P*
*TCGA*							
Risk score	Low/high	475/475	4.18 (2.50-6.97)	4.37*E* − 08	418/411	4.40 (2.31-8.39)	6.62*E* − 06
Age	≤50/>50	290/660	0.67 (0.43-1.03)	6.59*E* − 02	251/578	0.66 (0.39-1.11)	1.14*E* − 01
Pathologic stage	I-II/III-IV	723/216	3.34 (2.15-5.19)	8.81*E* − 08	645/184	4.28 (2.49-7.34)	1.31*E* − 07
ER status	N/P	214/697	0.49 (0.31-0.77)	1.78*E* − 03	191/638	1.35 (0.32-5.61)	6.82*E* − 01
PR status	N/P	304/604	0.46 (0.30-0.71)	5.34*E* − 04	274/555	0.56 (0.26-1.19)	1.29*E* − 01
HER2 status	N/P	680/160	0.93 (0.47-1.82)	8.22*E* − 01	671/158	0.72 (0.30-1.69)	4.47*E* − 01
Triple negative	No/yes	804/146	1.62 (0.97-2.71)	6.39*E* − 02	685/144	1.89 (0.43-8.35)	4.02*E* − 01
*GSE20685*							
Risk score	Low/high	164/163	2.41 (1.58-3.68)	4.16*E* − 05	164/158	2.22 (1.43-3.43)	3.55*E* − 04
Age	≤50/>50	209/118	0.74 (0.48-1.14)	1.71*E* − 01	206/116	0.79 (0.49-1.27)	3.36*E* − 01
T stage	I-II/III-IV	101/188	1.93 (1.50-2.49)	3.27*E* − 07	100/186	1.22 (0.86-1.74)	2.57*E* − 01
N stage	N0/N1-3	137/190	3.73 (2.26-6.15)	2.49*E* − 07	135/187	3.46 (1.99-6.01)	1.06*E* − 05
M stage	M0/M1	319/8	21.96 (10.49-45.99)	2.59*E* − 16	315/7	5.78 (2.02-16.58)	1.10*E* − 03
Adjacent CT	No/yes	54/268	2.07 (1.07-3.99)	3.05*E* − 02	54/268	1.34 (0.65-2.76)	4.26*E* − 01
*GSE21653*							
Risk score	Low/high	126/126	2.83 (1.77-4.53)	1.32*E* − 05	115/113	3.04 (1.80-5.11)	3.00*E* − 05
Age	≤50/>50	92/160	1.14 (0.72-1.79)	5.83*E* − 01	81/147	1.31 (0.79-2.17)	2.95*E* − 01
T stage	I-II/III-IV	178/66	1.70 (1.06-2.73)	2.75*E* − 02	164/64	1.20 (0.72-2.01)	4.87*E* − 01
N stage	N0/N1-3	116/133	1.54 (0.98-2.40)	5.84*E* − 02	105/123	1.61 (0.97-2.69)	6.81*E* − 02
ER status	N/P	110/140	0.66 (0.43-1.02)	5.94*E* − 02	100/128	0.46 (0.17-1.26)	1.31*E* − 01
PR status	N/P	124/126	0.84 (0.55-1.30)	4.35*E* − 01	114/114	1.43 (0.60-3.38)	4.15*E* − 01
HER2 status	N/P	207/26	1.59 (0.84-3.03)	1.58*E* − 01	204/24	1.09 (0.42-2.85)	8.60*E* − 01
Triple negative	No/yes	160/85	1.19 (0.74-1.89)	4.73*E* − 01	145/83	0.88 (0.26-2.90)	8.29*E* − 01
*GSE42568*							
Risk score	Low/high	52/52	3.68 (1.97-6.88)	4.55*E* − 05	51/50	4.18 (2.13-8.17)	2.99*E* − 05
Age	≤50/>50	27/77	0.66 (0.36-1.21)	1.79*E* − 01	25/76	0.72 (0.36-1.46)	3.63*E* − 01
N stage	N0/N1-3	45/59	4.35 (2.16-8.76)	3.88*E* − 05	44/57	4.72 (2.24-9.95)	4.53*E* − 05
ER status	N/P	34/67	0.44 (0.24-0.79)	6.35*E* − 03	34/67	0.40 (0.21-0.77)	5.77*E* − 03

HR: hazard ratio; CI: confidence interval; ER: estrogen receptor; PR: progesterone receptor; HER2/erbb2: epidermal growth factor receptor 2; Adjacent CT: adjacent chemotherapy; N: negative; P: positive.

**Table 2:** Details of the 11 transcription factors used to construct the prognostic signature.

Gene symbol	Gene stable ID	Gene type	Chr	Gene start (bp)	Gene end (bp)
E2F2	ENSG00000007968	Protein coding	1	23506438	23531233
EGR3	ENSG00000179388	Protein coding	8	22687659	22693480
EMX1	ENSG00000135638	Protein coding	2	72916260	72936071
FOXD1	ENSG00000251493	Protein coding	5	73444827	73448777
FOXJ1	ENSG00000129654	Protein coding	17	76136333	76141245
NKX6-1	ENSG00000163623	Protein coding	4	84491987	84498450
MSX1	ENSG00000163132	Protein coding	4	4859665	4863936
NR3C2	ENSG00000151623	Protein coding	4	148078762	148444698
PAX7	ENSG00000009709	Protein coding	1	18630846	18748866
STAT4	ENSG00000138378	Protein coding	2	191029576	191151596
ZNF552	ENSG00000178935	Protein coding	19	57803841	57814913

Abbreviation: Chr=chromosome.

## Data Availability

The datasets used and/or analyzed during the current study are available from the corresponding authors on reasonable request.

## Supplementary Materials

Supplementary Materials Supplementary Table S1: primer pairs for qPCR.
