# Supplementary material for: Identification of a Transcription Factor Signature That Can Predict Breast Cancer Survival
Source: Comput Math Methods Med. 2021 Feb 19;2021:2649123. doi: 10.1155/2021/2649123 (PMC7914092; doi:10.1155/2021/2649123)
Supplement: Supplementary Materials — Supplementary Table S1: primer pairs for qPCR. [file 2649123.f1.docx]

Table S1. Primer pairs for qPCR

| **TFs** | **Primer** | **Sequence** |
| --- | --- | --- |
| ACTB | Forward | CACCATTGGCAATGAGCGGTTC |
|  | Reverse | AGGTCTTTGCGGATGTCCACGT |
| E2F2 | Forward | CTCTCTGAGCTTCAAGCACCTG |
|  | Reverse | CTTGACGGCAATCACTGTCTGC |
| EGR3 | Forward | GACTCGGTAGTCCATTACAATCAG |
|  | Reverse | AGTAGGTCACGGTCTTGTTGCC |
| EMX1 | Forward | GCCTTCGAGAAGAACCACTACG |
|  | Reverse | CGGTTCTGGAACCACACCTTCA |
| FOXD1 | Forward | GATCTGTGAGTTCATCAGCGGC |
|  | Reverse | TGACGAAGCAGTCGTTGAGCGA |
| FOXJ1 | Forward | ACTCGTATGCCACGCTCATCTG |
|  | Reverse | GAGACAGGTTGTGGCGGATTGA |
| NKX6-1 | Forward | CCTATTCGTTGGGGATGACAGAG |
|  | Reverse | TCTGTCTCCGAGTCCTGCTTCT |
| NR3C2 | Forward | AAATCACACGGCGACCTGTCGT |
|  | Reverse | ATGGCATCCTGAAGCCTCATCC |
| PAX7 | Forward | GGAGGATGAAGCGGACAAGAAG |
|  | Reverse | AGGTCAGGTTCCGACTCCACAT |
| STAT4 | Forward | CAGTGAAAGCCATCTCGGAGGA |
|  | Reverse | TGTAGTCTCGCAGGATGTCAGC |
| ZNF552 | Forward | TGATGTGACGCTGGAGAACCTG |
|  | Reverse | GAGACACACCTGCCATAGGAGT |
| MSX1 | Forward | GACTCCTCAAGCTGCCAGAAGA |
|  | Reverse | ACGGTTCGTCTTGTGTTTGCGG |
